# SARS-CoV-2 Detection and Persistence in a Remote Amazonian Settlement

**DOI:** 10.1155/jotm/4872494

**Published:** 2025-12-02

**Authors:** Glauco M. Silva, Roberto C. Ilacqua, Franciely G. Gonçalves, Carla M. Santana, Felipe T. Jordão, Paula R. Prist, Melissa S. Nolan, Andreia F. Brilhante, Marcia A. Sperança, Gabriel Z. Laporta

**Affiliations:** ^1^Graduate Program in Health Sciences, FMABC University Center, Santo André, São Paulo, Brazil; ^2^Center for Health Sciences and Sports, Federal University of Acre, Rio Branco, State of Acre, Brazil; ^3^Center for Natural and Human Sciences, Federal University of ABC, São Bernardo do Campo, São Paulo, Brazil; ^4^Forests and Grasslands, IUCN, Washington, District of Columbia, USA; ^5^Arnold School of Public Health, University of South Carolina, Columbia, South Carolina, USA

**Keywords:** Amazon rainforest, COVID-19, rural health, SARS-CoV-2, spatial analysis

## Abstract

**Background:**

COVID-19 continues to pose a major global health challenge. Despite its geographic distance from Brazil's major urban centers, Acre state has experienced notable outbreaks. This study assessed the detection and persistence of SARS-CoV-2 in the rural settlement of Santa Luzia, located in the remote municipality of Cruzeiro do Sul, Acre state, Brazil.

**Methods:**

In July 2022, a cross-sectional survey was conducted at 40 sites from an ongoing environmental study, selected by deforestation patterns and proximity to health posts. Saliva samples were collected from residents aged 5–90 years, followed by nucleic acid extraction and multiplex RT-qPCR for SARS-CoV-2 detection.

**Results:**

Among the 183 individuals from 40 participating families, an 8% positivity rate was observed, with variation by age and sex. In 30% of families, at least one member tested positive, indicating continued viral presence within the community. Spatial analysis using Global and Local Moran's I statistics showed a random distribution of positive samples, consistent with multiple introductions from nearby urban centers and intermittent local persistence.

**Conclusion:**

These findings highlight the ongoing detection and persistence of SARS-CoV-2 in a remote Amazonian community, underscoring the need for continued surveillance in geographically isolated populations.

## 1. Introduction

COVID-19, caused by SARS-CoV-2, emerged globally in 2020, producing mostly mild to moderate illness but also severe cases and deaths [1–3]. By the end of the first year, over 80 million cases and nearly 2 million deaths were confirmed worldwide, with Brazil accounting for about 10% of both [4]. Acre, a state in Northern Brazil, despite its distance from major urban centers, experienced significant impacts, reporting 40,000 cases and 800 deaths in 2020 [4]. Bordered by Peru and Bolivia and largely covered by the Amazon rainforest, Acre faces logistical challenges due to limited road networks, seasonal flooding, and dependence on air and river transport [5, 6], contributing to its relative remoteness compared to other regions of Brazil [7–9].

**Figure 1:**
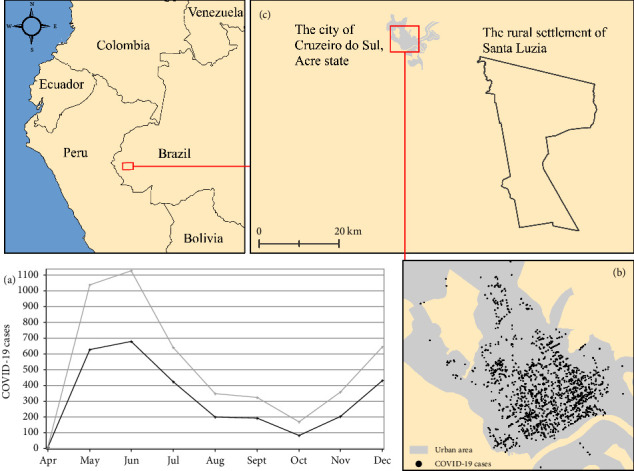
(a) Monthly time series of case incidence showing total cases (gray line) and georeferenced cases (black line) in 2020. (b) Georeferenced COVID-19 cases showing higher incidence rates in the urban area. (c) Study area, the rural settlement of Santa Luzia. ArcGIS v. 10.8.2 was utilized to create this map layout.

Cruzeiro do Sul, Acre's second-largest city, exemplifies these challenges [4, 10]. Its remoteness was historically relevant for observing viral spread in remote areas. In 2020, the municipality experienced a 5% incidence rate and a 1.7% case fatality ratio. The epidemic unfolded in three phases: a first wave (March–June), a decline (July–October), and a second wave (November-December) (Figure 1(a)). By georeferencing 2851 cases (61% of the total), a hotspot map was created, showing higher incidence rates in the urban areas of Cruzeiro do Sul (Figure 1(b)).

The observed cluster of COVID-19 in the urban area of Cruzeiro do Sul is unsurprising, as urban settings with higher population density, increased mobility, and cultural factors are more conducive to virus transmission [10]. However, SARS-CoV-2 also reached remote rural zones. Many non-georeferenced cases (*n* = 1810) lacked complete address details or were in rural locations with mismatched locality names [11]. This suggests that SARS-CoV-2 likely spread into the rural settlement of Santa Luzia in 2020 (Figure 1(c)).

This study aimed to assess SARS-CoV-2 detection and persistence in the Santa Luzia settlement following mass immunization campaigns in 2021-2022. We conducted a cross-sectional survey in July 2022 to determine the extent of viral detection among residents, describe its spatial distribution across households, and provide baseline evidence for continued surveillance in remote Amazonian communities.

## 2. Materials and Methods

### 2.1. Ethics

The study protocol received ethical clearance from the Centro Universitário Uninorte Ethics for Research Committee (opinion number 3.635.212/CAAE 20708819.3.0000.8028). The study was conducted in accordance with ethical principles for this type of research, and written informed consent was obtained from all participants. For children aged 5 years and older and teenagers under 18, written informed consent was obtained from both the participants and their parents. Children under 5 years old were not included in the study.

### 2.2. Study Area

**Figure 2:**
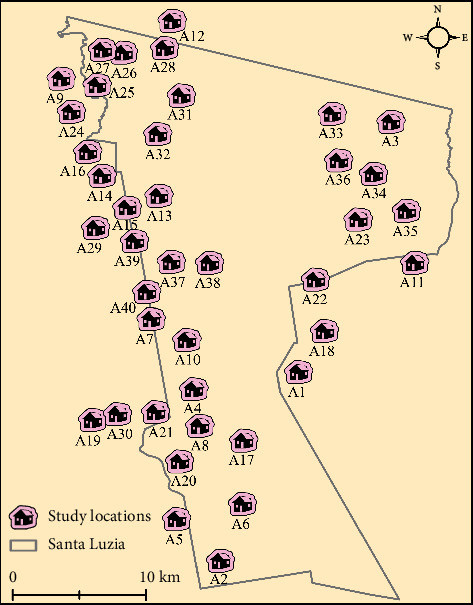
Study sites in the Santa Luzia rural settlement (2022), where families live in wooden-framed Amazonian houses without sewage or drinking water but with electricity, unpaved road access, and rural livelihoods. ArcGIS v. 10.8.2 was utilized to create this map layout.

Since the late 1970s, over 3700 rural agricultural settlements have been established in Brazil, covering more than 75 million hectares [12]. Managed by INCRA, these settlements consist of land redistributed to families lacking rural property options [13]. In 2022, we launched a research program to investigate community health and SARS-CoV-2 detection in 40 landscape sites within the rural settlement of Santa Luzia (Figure 2).

### 2.3. Sampling

Locations were selected using a convenience sampling approach. The 40 sites (Figure 2) correspond to lots originally established for environmental and land-use monitoring, chosen according to deforestation history, forest cover, and proximity to local health posts [14]. This sample was designed to assess SARS-CoV-2 presence and point prevalence. Each site represents an INCRA lot inhabited by a consenting family, primarily engaged in cassava cultivation and pasture management [15]. The settlement's native tropical moist forest, with clay-sand dystrophic yellow Argissolo (Acrisol) soil, supports year-round water availability due to a high water table [16], while non-forest areas are mainly pasturelands [17].

### 2.4. Design and Collection

In July 2022, we conducted a cross-sectional survey in the 40 locations (Figure 2). Saliva samples were self-collected from participants aged 5–90 years, following informed consent [18]. Each participant received a sealed envelope with collection instructions, an ID form, a sterile cotton swab, a tube for storage, and an isopropanol-soaked tissue. After moistening the swab in their mouth for one minute, participants placed it in the tube, sealed it, and returned it. Samples were stored at −20°C until laboratory analysis.

### 2.5. RNA Extraction From Saliva

For nucleic acid extraction, 900 μL of Trizol reagent was added to the saliva sample, and the cotton swab was pressed to release the sample. The mixture was then transferred to a tube with 250 μL of chloroform, shaken, and centrifuged at 20, 000 × g for 5 min at 40°C. The aqueous phase was transferred to a new tube with 900 μL of ethanol and centrifuged for 15 min. After removing the ethanol, the pellet was washed with 70% ethanol, dried, and dissolved in 40 μL of 10 mM Tris-HCl (pH 7.5–8.0).

### 2.6. Diagnosis of SARS-CoV-2

SARS-CoV-2 diagnosis was performed using the Allplex SARS-CoV-2 Assay by Seegene Brazil, which detects four target genes: RdRP, S protein, N protein, and the E protein for all *Sarbecovirus*. Two microliters of the sample were used, with water as the negative control. The RT-qPCR reactions were conducted on the BioRad CFx96, with cycling conditions of 20 min at 50°C, 15 min at 95°C, and 45 cycles at 95°C for 15 s, 60°C for 10 s, and 72°C for 15 s. Samples were considered positive if any of the molecular targets were detected.

### 2.7. Spatial Analysis

To investigate the spatial distribution of SARS-CoV-2 cases in Santa Luzia, we analyzed household-level data using Global and Local Moran statistics [19]. The Global Moran index measured overall spatial autocorrelation, determining if cases were clustered, dispersed, or randomly distributed. The Local Moran index identified specific clusters (high-high or low-low) and outliers (high-low or low-high). These analyses describe the spatial occurrence of SARS-CoV-2 detection across households.

## 3. Results

### 3.1. SARS-CoV-2 Detection and Positivity Rate

All 40 selected families participated in the study, supported by trusted public health officials from Cruzeiro do Sul. A total of 183 individuals consented to SARS-CoV-2 testing via self-collected saliva samples, with 15 (8%) testing positive. Positivity rates varied by group: boys/teens 5% (2/38), men 12% (5/43), girls/teens 16% (5/31), and women 4% (3/71). SARS-CoV-2 was detected in 12 households (30%), mostly with a single case, except for two households with multiple cases, including parent–child pairs (Table 1).

### 3.2. Viral RNA Detection

**Figure 3:**
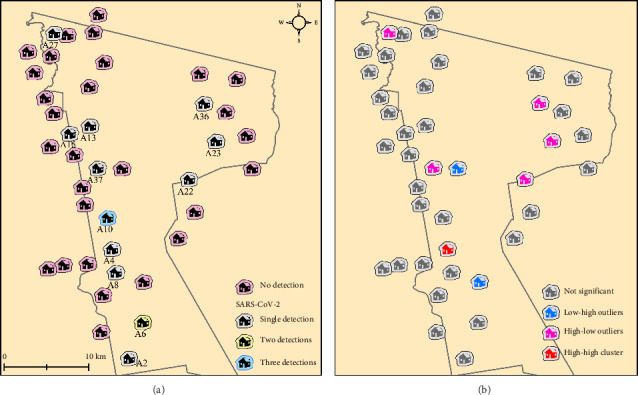
(a) SARS-CoV-2 detections among households. (b) Cluster analysis results using Local Moran's index. ArcGIS v. 10.8.2 was utilized to create this map layout.

Figure 3(a) shows a household with three positive members (a mother and her sons) exhibiting standard cycle threshold values (∼30 cycles) for all three SARS-CoV-2 targets in the saliva. Most other positive cases showed one or two targets, mainly the N gene, with higher Ct values (∼35 cycles), reflecting lower viral RNA levels.

### 3.3. Spatial Distribution

Figure 3(b) shows that positive samples do not form a localized cluster within households. The spatial pattern indicates that SARS-CoV-2 detection was distributed across households without a clear clustering pattern, highlighting persistence of viral RNA in multiple locations within the settlement.

## 4. Discussion

Our findings show that SARS-CoV-2 RNA was detectable in households in the rural settlement of Santa Luzia by 2022, following earlier circulation in the nearby urban center of Cruzeiro do Sul. Detection occurred across multiple households, with no evidence of spatial clustering, and most positive samples had low viral RNA levels.

We discuss three main aspects: (1) factors influencing SARS-CoV-2 presence in the settlement; (2) broader Amazonian context, including cross-border comparisons; and (3) utility of saliva-based RT-qPCR for surveillance in remote settings.

### 4.1. Factors Influencing SARS-CoV-2 Presence

Santa Luzia was established in 1982 amid a broader shift from rubber extraction to agriculture and livestock production in Acre [15, 17]. Government policies in the 1980s promoted family farming on former rubber plantations, enhancing economic activity and access to education and health services [20]. Today, residents transport and sell agricultural goods including meat, cassava flour, fish, and coffee in nearby urban markets, facilitated by the BR-364 highway [21]. These interactions may have facilitated repeated introductions of SARS-CoV-2 into the settlement.

Similar patterns have been observed in isolated Amazonian indigenous communities in Ecuador, where urban connections and market travel contributed to high infection rates despite voluntary isolation measures [22, 23].

### 4.2. Broader Amazonian Context

Our results align with trends observed across urban and remote Amazonian communities. In Belém (Pará), seroprevalence reached 39% after the first wave [24], and in Manaus (Amazonas), seroprevalence reached 76%, reflecting a largely unmitigated epidemic with high mortality [25]. Remote Indigenous and Quilombola populations also showed high seroprevalence (up to 73% and 41%, respectively) [26–28]. Additional studies reported long-term COVID-19 impacts [29, 30] and SARS-CoV-2 variants associated with severe disease in Indigenous populations [31].

Beyond Brazil, SARS-CoV-2 detection in rural and Indigenous communities in the Ecuadorian Amazon mirrors our findings, with attack rates of 26%–90% despite isolation strategies [22, 32]. These studies also documented high viral loads in a subset of community members, comparable to the variations observed in our dataset.

Taken together, these cross-border examples emphasize that geographic remoteness does not prevent viral persistence and highlight the relevance of social structure, mobility, and limited healthcare access across the Amazon basin.

### 4.3. Saliva-Based RT-qPCR for Remote Surveillance

Surveillance in rural areas requires accurate, low-cost, and logistically feasible diagnostics. Standard nasopharyngeal testing with −80°C storage is impractical in such settings [33]. Saliva-based RT-qPCR provides a practical alternative, compatible with −20°C storage and transport at room temperature [18].

Data from the Ecuadorian Amazon emphasize that accessible testing is critical, as isolation alone did not prevent outbreaks [23]. Saliva-based RT-qPCR is well suited for settings with unstable power and limited cold-chain infrastructure [34, 35]. Beyond SARS-CoV-2, saliva-based diagnostics have been successfully applied to detect *Plasmodium falciparum* in Cameroon and Zambia [36] and *Trypanosoma cruzi* and *Leishmania braziliensis* in Brazil [37, 38]. These methods could strengthen surveillance for multiple endemic pathogens in rural Amazonian communities.

### 4.4. Limitations

A limitation of our study is the lack of confirmatory nasopharyngeal swabs. However, the saliva-based assay has been validated for field detection of SARS-CoV-2 RNA [18]. The convenience sampling limits statistical inference but was sufficient to assess viral presence and persistence.

## 5. Conclusion

Our study assessed SARS-CoV-2 presence and persistence in a remote Amazonian settlement post-immunization (2021-2022). Viral RNA was detected across multiple households, with no clear spatial clustering, and most samples showed low RNA levels. Saliva-based RT-qPCR proved effective for detecting SARS-CoV-2 in this low-resource setting. Overall, this study highlights its potential for broader surveillance of endemic and emerging pathogens across the Amazon basin.

## Figures and Tables

**Table 1:** SARS-CoV-2 detection by age and sex in each sampling unit, Santa Luzia, 2022.

Locations	Boys or teens *n*. detected/total	Men *n*. detected/total	Girls or teens *n*. detected/total	Women *n*. detected/total
A1	0/1	0/1	0	0/1
A2	0	1/2	0/2	0/4
A3	0/1	0/1	0/2	0/2
A4	0/1	1/2	0	0/4
A5	0/4	0/1	0	0/1
A6	0/1	1/1	1/2	0/1
A7	0/1	0/2	0	0/1
A8	0	0/1	0	1/2
A9	0/1	0/1	0/1	0/2
A10	2/2	0	0	1/1
A11	0	0	0	0/2
A12	0	0	0	0/2
A13	0/1	0/1	1/3	0/4
A14	0	0/1	0/2	0/2
A15	0/2	1/1	0	0/3
A16	0/2	0/1	0/1	0/1
A17	0/4	0/2	0/2	0/2
A18	0/2	0	0	0/1
A19	0	0/1	0/1	0/1
A20	0	0/1	0	0/1
A21	0	0/1	0	0/1
A22	0/1	0/3	1/2	0/1
A23	0/2	0/1	1/2	0/2
A24	0/2	0/2	0/2	0/4
A25	0	0/2	0	0/2
A26	0/3	0/1	0/1	0/1
A27	0	0/1	0	1/3
A28	0	0/1	0	0/1
A29	0/3	0/2	0/1	0/3
A30	0	0/1	0/1	0/3
A31	0/1	0	0/2	0
A32	0	0/1	0	0/2
A33	0/1	0/1	0	0
A34	0	0/1	0	0/2
A35	0/2	0	0/1	0/2
A36	0	1/1	0/1	0
A37	0	0/1	1/1	0/1
A38	0	0	0/1	0/2
A39	0	0/2	0	0/1
A40	0	0/1	0	0/2

## Data Availability

The data that support the findings of this study are available from the corresponding author upon reasonable request.

## References

[1] Andersen K. G., Rambaut A., Lipkin W. I., Holmes E. C., Garry R. F. (2020). The Proximal Origin of SARS-CoV-2. *Nature Medicine*.

[2] Wu J. T., Leung K., Bushman M. (2020). Estimating Clinical Severity of COVID-19 From the Transmission Dynamics in Wuhan, China. *Nature Medicine*.

[3] Huang C., Wang Y., Li X. (2020). Clinical Features of Patients Infected With 2019 Novel Coronavirus in Wuhan, China. *The Lancet*.

[4] Johns Hopkins University COVID-19 Data Repository by the Center for Systems Science and Engineering. https://github.com/CSSEGISandData/COVID-19.

[5] Castiglioni L., Bicudo H. E. M. D. C. (2005). Molecular Characterization, Relatedness of *Haematobia irritans* (Horn Fly) Populations, by RAPD-PCR. *Genetica*.

[6] Rocha R. D. C., Cardoso A. D. S., Souza J. L. D. (2023). First Official Record of Aedes (Stegomyia) Albopictus (Diptera: Culicidae) in the Acre State, Northern Brazil. *Revista do Instituto de Medicina Tropical de São Paulo*.

[7] de Oliveira Padilha M. A., de Oliveira Melo J., Romano G. (2019). Comparison of Malaria Incidence Rates and Socioeconomic-Environmental Factors Between the States of Acre and Rondônia: A Spatio-Temporal Modelling Study. *Malaria Journal*.

[8] Gonçalves F. G., Belone A. D. F. F., Rosa P. S., Laporta G. Z. (2019). Underlying Mechanisms of Leprosy Recurrence in the Western Amazon: A Retrospective Cohort Study. *BMC Infectious Diseases*.

[9] Lana R. M., Gomes M. F. D. C., Lima T. F. M. D., Honório N. A., Codeço C. T. (2017). The Introduction of Dengue Follows Transportation Infrastructure Changes in the State of Acre, Brazil: A Network-Based Analysis. *PLoS Neglected Tropical Diseases*.

[10] Lima De Assis E., José de Deus Morais M., De Oliveira Eichemberg J. (2021). Evolution of COVID-19 During the Epidemiological Week 16 to 53 of 2020 in the State of Acre Western Amazonia, Brazil. *Journal of Human Growth and Development*.

[11] Silva G. M. (2023). *Spatial Evolution of the First Wave of COVID-19 in 2020 in the Municipality of Cruzeiro Do Sul, AC, Brazil (PhD Thesis)*.

[12] Pereira A. S. A. D. P., Dos Santos V. J., Alves S. D. C. (2022). Contribution of Rural Settlements to the Deforestation Dynamics in the Legal Amazon. *Land Use Policy*.

[13] Laporta G. Z., Ilacqua R. C., Bergo E. S. (2021). Malaria Transmission in Landscapes With Varying Deforestation Levels and Timelines in the Amazon: A Longitudinal Spatiotemporal Study. *Scientific Reports*.

[14] Laporta G. Z., Valle D., Prist P. R. (2025). Intermediate Forest Cover and Malaria Risk in an Amazon Deforestation Frontier. *Acta Tropica*.

[15] Ludewigs T., Brondizio E. S. (2009). Paths of Diversification: Land Use, Livelihood Strategies and Social Learning Along the Aging of a Land Reform Settlement in Acre, Brazil. *Amazônica-Revista de Antropologia*.

[16] da Silva Abel E. L., Delgado R. C., Vilanova R. S. (2021). Environmental Dynamics of the Juruá Watershed in the Amazon. *Environment, Development and Sustainability*.

[17] Salisbury D. S., Schmink M. (2007). Cows Versus Rubber: Changing Livelihoods Among Amazonian Extractivists. *Geoforum*.

[18] De Oliveira L. P. R., Cabral A. D., Dos Santos Carmo A. M. (2022). Alternative SARS-CoV-2 Detection Protocol From Self-Collected Saliva for Mass Diagnosis and Epidemiological Studies in Low-Incoming Regions. *Journal of Virological Methods*.

[19] Soares T. S. M., Latorre M. D. R. D. D. O., Laporta G. Z., Buzzar M. R. (2010). Análise Espacial e Sazonal da Leptospirose no Município de São Paulo, SP, 1998 a 2006. *Revista de Saúde Pública*.

[20] Peralta P., Mather P. (2000). An Analysis of Deforestation Patterns in the Extractive Reserves of Acre, Amazonia From Satellite Imagery: A Landscape Ecological Approach. *International Journal of Remote Sensing*.

[21] Redwood J. (2012). *Managing the Environmental and Social Impacts of Major IDB-Financed Road Improvement Projects in the Brazilian Amazon: The Case of BR-364 in Acre*.

[22] Henriquez-Trujillo A. R., Ortiz-Prado E., Rivera-Olivero I. A. (2021). COVID-19 Outbreaks Among Isolated Amazonian Indigenous People, Ecuador. *Bulletin of the World Health Organization*.

[23] Ortiz-Prado E., Rivera-Olivero I. A., Freire-Paspuel B. (2021). Testing for SARS-CoV-2 at the Core of Voluntary Collective Isolation: Lessons From the Indigenous Populations Living in the Amazon Region in Ecuador. *International Journal of Infectious Diseases*.

[24] da Silva Torres M. K., Lopes F. T., de Lima A. C. R. (2022). Seroprevalence and Risk Factors for COVID-19 in the Metropolis of the Brazilian Amazon. *Scientific Reports*.

[25] Buss L. F., Prete C. A., Abrahim C. M. M. (2021). Three-Quarters Attack Rate of SARS-CoV-2 in the Brazilian Amazon During a Largely Unmitigated Epidemic. *Science*.

[26] Lima C. N. C., Abreu I. N., Rodrigues E. P. S. (2022). Anti-SARS-CoV-2 Antibodies Among Indigenous Populations of the Brazilian Amazon: A Cross-Sectional Study. *BMJ Open*.

[27] Pereira K. A. S., de Lima L. N. F., Botelho B. J. S. (2025). Socioecology and Prevalence of SARS-CoV-2 Infection in Quilombolas Living in the Brazilian Amazon. *American Journal of Human Biology*.

[28] Rodrigues E. P. S., Abreu I. N., Lima C. N. C. (2021). High Prevalence of Anti-SARS-CoV-2 IgG Antibody in the Xikrin of Bacajá (Kayapó) Indigenous Population in the Brazilian Amazon. *International Journal for Equity in Health*.

[29] Galúcio V. C. A., De Menezes D. C., Chaves E. C. R. (2024). Laboratory Profiling of Patients With Long COVID in the Brazilian Amazon Region: A Cross‐Sectional Study. *Journal of Medical Virology*.

[30] Miranda A. L. D. C., Costa V. L. D. S., da Paixão A. R. T. (2024). Factors Associated With Access to Health Services Among People With Long COVID in the Brazilian Amazon. *Frontiers in Public Health*.

[31] Coelho R. D. C. C., Martins C. L. E. L. P., Pastana L. F. (2024). Molecular Profile of Variants Potentially Associated With Severe Forms of COVID-19 in Amazonian Indigenous Populations. *Viruses*.

[32] Morales-Jadán D., Vallejo-Janeta A. P., Bastidas V. (2023). High SARS-CoV-2 Infection Rates and Viral Loads in Community-Dwelling Individuals From Rural Indigenous and Mestizo Communities From the Andes During the First Wave of the COVID-19 Pandemic in Ecuador. *Frontiers of Medicine*.

[33] Poulakou G., Barakat M., Israel R. J., Bacci M. R. (2023). Ribavirin Aerosol in Hospitalized Adults With Respiratory Distress and COVID‐19: An Open‐Label Trial. *Clinical and Translational Science*.

[34] Lin S. C., Hsu M. Y., Kuan C. M. (2014). Cotton-Based Diagnostic Devices. *Scientific Reports*.

[35] Cheng C. M., Kuan C. M., Chen C. F. (2016). *Vitro Diagnostic Devices*.

[36] Tao D., McGill B., Hamerly T. (2019). A Saliva-Based Rapid Test to Quantify the Infectious Subclinical Malaria Parasite Reservoir. *Science Translational Medicine*.

[37] De Oliveira L. C., Pereira N. B., Moreira C. H. V. (2020). ELISA Saliva for *Trypanosoma cruzi* Antibody Detection: An Alternative for Serological Surveys in Endemic Regions. *The American Journal of Tropical Medicine and Hygiene*.

[38] Brito M. E. F. D., Almeida E. L., Medeiros A. C. R. (2018). Leishmania (Viannia) Braziliensis Isolated From the Saliva of Patients in a Cutaneous Leishmaniasis-Endemic Area of Northeastern Brazil. *Memórias do Instituto Oswaldo Cruz*.

